# Conformational Flexibility of a Short Loop near the Active Site of the SARS-3CLpro is Essential to Maintain Catalytic Activity

**DOI:** 10.1038/srep20918

**Published:** 2016-02-16

**Authors:** Chunmei Li, Xin Teng, Yifei Qi, Bo Tang, Hailing Shi, Xiaomin Ma, Luhua Lai

**Affiliations:** 1 BNLMS, State Key Laboratory for Structural Chemistry of Unstable and Stable Species, College of Chemistry and Molecular Engineering, Peking University, Beijing, 100871, China; 2 Center for Quantitative Biology, Peking University, Beijing, 100871, China

## Abstract

The SARS 3C-like proteinase (SARS-3CLpro), which is the main proteinase of the SARS coronavirus, is essential to the virus life cycle. This enzyme has been shown to be active as a dimer in which only one protomer is active. However, it remains unknown how the dimer structure maintains an active monomer conformation. It has been observed that the Ser139-Leu141 loop forms a short 3_10_-helix that disrupts the catalytic machinery in the inactive monomer structure. We have tried to disrupt this helical conformation by mutating L141 to T in the stable inactive monomer G11A/R298A/Q299A. The resulting tetra-mutant G11A/L141T/R298A/Q299A is indeed enzymatically active as a monomer. Molecular dynamics simulations revealed that the L141T mutation disrupts the 3_10_-helix and helps to stabilize the active conformation. The coil-3_10_-helix conformational transition of the Ser139-Leu141 loop serves as an enzyme activity switch. Our study therefore indicates that the dimer structure can stabilize the active conformation but is not a required structure in the evolution of the active enzyme, which can also arise through simple mutations.

The first outbreak of severe acute respiratory syndrome (SARS) occurred over 10 years ago in 2003. This outbreak has led to significant attention and intensive study of the treatment and prevention of coronaviruses over the past decade. Recently, a new coronavirus called the Middle East Respiratory Syndrome Coronavirus (MERS-CoV) has emerged. MERS-CoV first appeared in Saudi Arabia in 2012 and quickly spread into Europe. To date, approximately 3-4 out of every 10 patients who have been diagnosed with MERS have died^1^. Although significant advances in the understanding of coronaviruses have been made over the past decade through the study of SARS-CoV, more research is needed to develop effective countermeasures, such as drugs or vaccines, to control the new pathogen.

The 3C-like proteinase (3CLpro) has received much attention as a potential key anti-CoV target^2^. Like other known CoV-3CLpro structures, such as TGEV, hCoV-229E, hCoV-HKU1, and IBV^2^,^3^,^4^,^5^, SARS-3CLpro has a highly conserved three-dimensional structure, dimer interface, catalysis dyad, and substrate binding site, but an extremely low homology with cellular proteases. SARS-3CLpro forms a homodimer in crystal structures^6^,^7^ that consists of two protomers oriented almost perpendicularly. Each protomer of the SARS-3CLpro contains three domains. Domains I (residues 1-101) and II (residues 102-184) have trypsin-like folds and control catalysis. Domain III (residues 201-306) is an extra domain with five α-helices that is related to enzyme dimerization and activity regulation^8^. The substrate binding site is located in the cleft between Domains I and II, which is composed of six subsites (S1-S6) corresponding to the P1-P6 peptides of the substrate peptide. S1 is the most important subsite, because residue Gln-P1 of this subsite is conserved in all coronaviruses.

SARS-3CLpro exists in a monomer-dimer equilibrium in solution, but only the dimer is active^9^. The N-finger α-helix A’ of Domain I (residues 10-15) and helix Domain III have been recognized as the main components of the dimer formation. Changes in these components are known to disrupt the monomer-dimer equilibrium. For example, the proteinase loses its activity and remains monomeric in solution when the N-finger is deleted^10^. Mutations of either Gly11 or Arg298 to Ala also lead to inactive monomers^11^,^12^. The G11A mutation may shorten the α-helix A’ of Domain I, which in turn may disrupt the N-finger conformation, preventing the N-finger from correctly squeezing into the pocket of another monomer during dimerization and leading to the monomer structure^11^. Similarly, the R298A mutation may trigger the switch from a dimer to a monomer by releasing the constraints between the N-finger and C terminus and destroying their precise positioning and orientation^12^. Ser139 and Phe140 are two key residues that not only contribute to interactions between the two protomers in the parent dimer but also maintain the correct conformation of the S1 subsite in the substrate-binding pocket. Although Ser139 and Phe140 are adjacent, their mutations cause different conformational changes in the crystal structures^13^. The S139A mutation forms monomers in the crystal structure but maintains partial activity in solution due to the existence of a small fraction of dimers in solution. In contrast, the F140A mutation forms dimers with a highly collapsed substrate-binding pocket and is totally inactive. The active site mutation, C145A, loses hydrolysis activity completely but remains a dimer^14^. Other residue mutations, which are neither on the dimer interface nor key to catalysis, can also influence enzyme activity and dimer association-dissociation of SARS-3CLpro via long-range interactions^15^,^16^.

All current experimental and computational evidence supports the hypothesis that the dimer structure is necessary for SARS-3CLpro activity^17^. Meanwhile, all mutants that disrupt the dimer structure are inactive^10^,^11^,^12^,^13^. However, the conformational characteristics in the active protomer that are essential to catalysis remain to be determined. To the best of our knowledge, no active monomer mutant has been reported previously for SARS-3CLpro or the main proteinases of other CoVs. In the present study, we successfully designed an active monomer of SARS-3CLpro by comparing the active and inactive structures of the proteinase. We studied the mechanism that regulates this active monomer using mutational and enzymatic studies, as well as molecular dynamics simulations.

## Results

### Active monomer design

**Figure 1:**
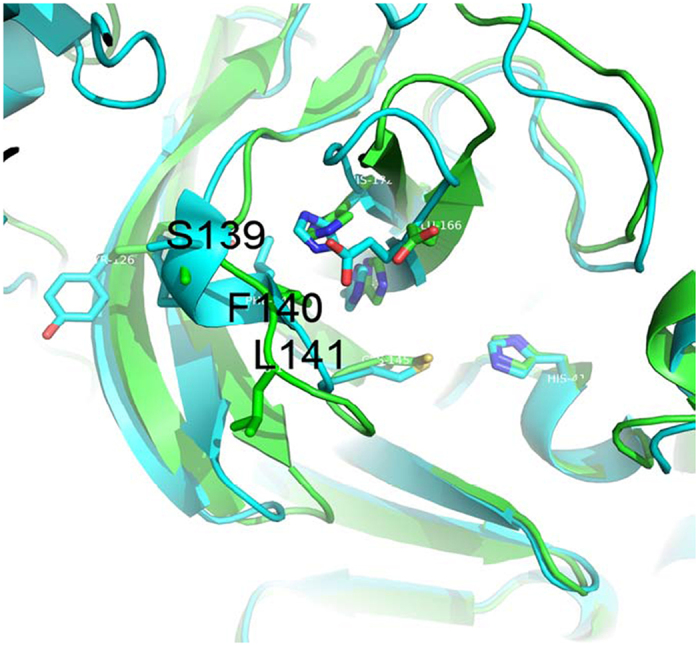
Conformational differences between the active protomer (protomer A) of SARS-3CLpro and the R298A mutant (drawn by PYMOL1.4.1). The active protomer of SARS-3CLpro (PDB ID 1UK2) and the R298A mutant (PDB ID 2QCY) are shown in green and cyan, respectively. The most distinguishable characteristic of the R298A mutant is the formation of a short 3_10_-helix instead of an active-site loop in residues Ser139-Phe140-Leu141.

By comparing the active protomer (protomer A) of the wild-type SARS-3CLpro with both the inactive protomer (protomer B) of the wild-type SARS-3CLpro (PDB Code 1UK2)^6^ and the inactive monomer R298A (PDB Code 2QCY)^12^, we found that the most distinguishable differences between the active and inactive structures were in the conformational changes of residues Ser139-Phe140-Leu141. Ser139-Phe140-Leu141 is the part of the oxyanion loop that participates in maintaining the correct conformation of the S1 subsite in the active enzyme. In contrast, in inactive enzymes, this short peptide forms a short 3_10_-helix that twists the oxyanion hole of the S1 subsite (Fig. 1). This same conformational change is also observed in the collapsed 3CLpro of avian infectious bronchitis virus (IBV-3CLpro)^5^.

Formation of a 3_10_-helix in inactive structures implies that disruption of this 3_10_-helix will make the structure more flexible, and may produce an active monomer. Among the three residues in the 3_10_-helix, Leu141 is a strong helix former^18^,^19^. We hypothesized that changing Leu141 to another residue with a lower helical tendency and strong β-structure tendency may destroy the 3_10_-helix. In the present work, we mutated Leu141 to Thr141, a residue with strong β-structure and low helix tendency. Simultaneously, we mutated Gly11, Arg298, and Gln299, which are residues at the dimer interface, to Ala^8^,^11^,^15^ to generate a stable monomeric protein. The triple-mutant G11A/R298A/Q299A (GRQ) was confirmed to be a monomer, and the tetra-mutant G11A/L141T/R298A/Q299A (GLRQ) was a monomer with enzyme activity as designed.

### Enzyme Activity of GLRQ

**Figure 2:**
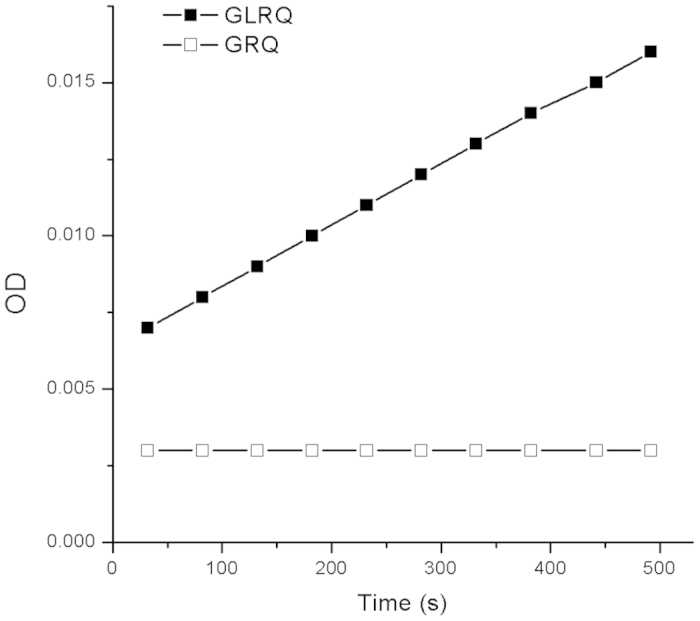
The enzyme activity of GRQ and GLRQ. Equal concentrations of GRQ and GLRQ (18 μM) were reacted with 200 μM of the colorimetric substrate, Thr-Ser-Ala-Val-Leu-Gln-pNA, at 37 °C. The symbol, ―□―, represents the enzyme activity of GRQ, and ―■― represents the enzyme activity of GLRQ.

Both GRQ and GLRQ were incubated with a pNA substrate (Thr-Ser-Ala-Val-Leu-Gln-pNA) at a concentration of 200 μM at 37 °C. GRQ exhibited no enzyme activity. After introducing L141T, however, GLRQ was shown to have regained hydrolytic activity (Fig. 2). To determine the amount of activity recovered in GLRQ, the reaction rates of equal amounts of GLRQ and wild-type 3CLpro (as a control) were measured (Fig. S1). Compared with 3CLpro, GLRQ regained 2% of proteolysis activity.

We derived *k*_*cat*_ and *K*_*m*_ of GLRQ using the Lineweaver-Burk plot with a fixed enzyme concentration and a series of substrate concentrations. *K*_*m*_ of GLRQ was 0.68 ± 0.04 mM and *k*_*cat*_ was 0.09 ± 0.001 min^−1^. In comparison, *K*_*m*_ is 0.98 ± 0.36 mM and *k*_*cat*_ is 6.4 ± 2.0 min^−1^ in the wild-type enzyme^20^. Thus, GLRQ and 3CLpro have similar binding affinity, whereas catalytic activity is 70 times lower and *k*_*cat*_/*K*_*m*_ is 50 times lower in GLRQ compared with the wild-type enzyme. This finding demonstrates that introducing Thr at position 141 to disrupt the 3_10_-helix can indeed recover proteolysis activity in GLRQ.

### Oligomeric state of GLRQ

Analytical gel filtration and sedimentation velocity methods were used to check whether both the GRQ and GLRQ mutants maintain monomer structure. The same concentration of wild-type 3CLpro served as a control.

**Figure 3:**
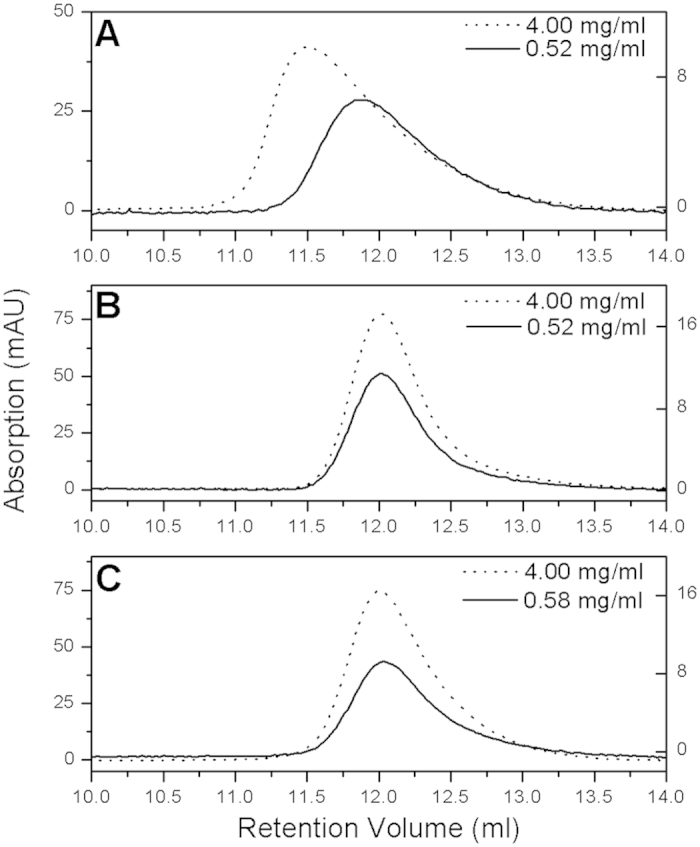
Oligomeric states of 3CLpro (**A**), GRQ (**B**), and GLRQ (**C**) on an analytic gel filtration column. Proteins with low concentrations (0.52 mg/ml for 3CLpro and GRQ, 0.58 mg/ml for GLRQ) are indicated by a solid line and proteins with high concentrations (4.0 mg/ml) are indicated by a dotted line.

The Superdex 75 HR 10/300 GL column (GE Healthcare, Pittsburgh, PA, USA) was used for gel filtration analysis. A standard calibration curve was fitted according to the retention volume of known markers (Supplemental Table S1, Fig. S2). GRQ and GLRQ appeared as a single peak, and the maximum retention volume did not change with the concentration, indicating that only one species was present in solution. For 3CLpro, the peak shifted to the high molecular weight side as the concentration increased, indicating the existence of subunit exchange (Fig. 3). The retention volumes for GRQ and GLRQ were all approximately 12 ml at both high and low concentrations, corresponding to the molecular weight of a monomer (Table S2).

**Figure 4:**
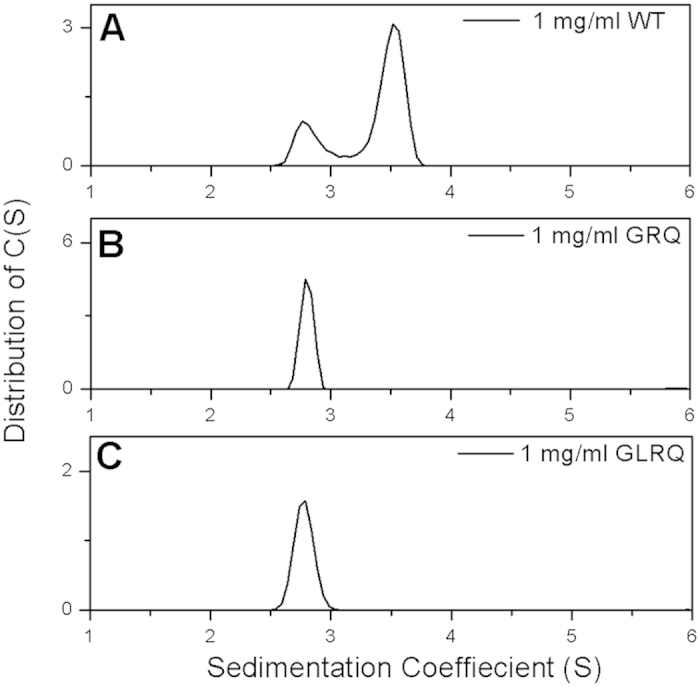
The sedimentation coefficient distribution of 3CLpro (**A**), GRQ (**B**), and GLRQ (**C**). 3CLpro exhibits both a monomer and dimer peak at 1 mg/ml, but GRQ and GLRQ exhibit only a monomer peak at the same concentration.

A sedimentation velocity experiment was conducted at 20 °C in PBS with 1 mg/ml concentrations for 3CLpro, GRQ, and GLRQ to further verify the oligomeric state of the GLRQ mutant. A single peak was observed for both GRQ and GLRQ at approximately 2.7 s (Fig. 4), which corresponds to a monomer^10^. Two peaks were observed at the monomer and dimer positions, respectively, consistent with previous reports^10^,^21^.

Previous studies have shown that dimer formation can be induced or enhanced by either substrate or substrate-analogue (**5f**) binding^21^,^22^. **5f** has a similar binding pattern to the enzyme as the substrate, and was used as a probe to test whether the active pocket of the SARS-3CLpro adopts the correct conformation. Under our experimental condition, 18.5 μM GLRQ was totally inhibited by 160 μM **5f** (Supplemental Fig. S3), which implies that the substrate binding pocket of the GLRQ mutant maintains the active conformation. In addition, when 10 μM **5f** was incubated with 5 μM GLRQ in PBS and analysed using the sedimentation velocity experiment described above, no peaks were induced except for the monomer peak (Supplemental Fig. S4). This finding further supports that the GLRQ mutant is monomeric in solution and does not dimerize in the presence of the substrate or its analogue.

### Enzyme activity of GLRQ and its dependence on enzyme concentration

**Figure 5:**
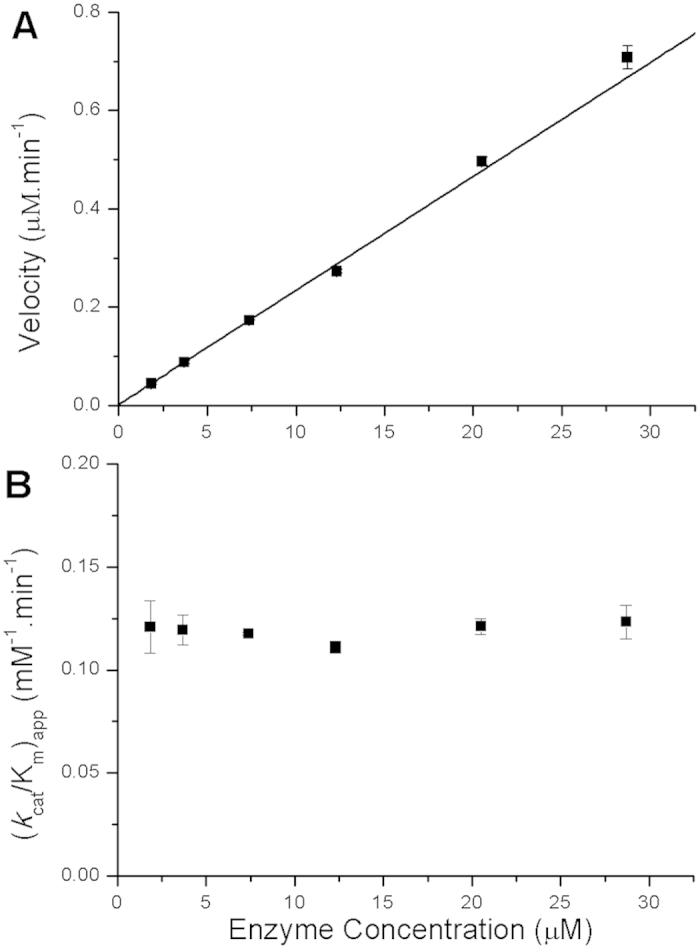
Enzyme activity dependence of GLRQ at different concentrations. (**A**) Velocity versus enzyme concentration. (**B**) *k*_cat_/*K*_*m*_ versus enzyme concentration. Velocity increases in a linear manner with enzyme concentration when the concentration of the peptide-pNA substrate is maintained at 200 μM; *k*_cat_/*K*_*m*_does not change.

SARS-3CLpro exists as a monomer-dimer equilibration in solution, and the dimer is the active form of the proteinase. Its enzymatic-specific activity, *k*_cat_/*K*_m_, increases with an increase in enzyme concentration^9^,^21^,^22^, which provides evidence that further dimerization can be induced by the substrate during hydrolysis. Furthermore, a previous study has shown upward curvature in the reaction rate versus enzyme concentration plots for 3CLpro^23^. We measured the enzyme activity of GLRQ at different enzyme concentrations, and found that the velocity increases in a linear manner with an increase in enzyme concentration, which is in contrast to the activity of the wild-type enzyme (Fig. 5A). Although GLRQ hydrolyses the colorimetric pNA substrate with a lower efficiency than the wild-type proteinase, the reaction rate increases proportionally with a positive slope that goes through the origin; i.e., *k*_cat_/*K*_*m*_ did not change with enzyme concentration (Fig. 5B). This result indicates that the enzyme is active as a monomer with no occurrence of substrate-induced dimerization.

### Molecular dynamics simulations of GLRQ

To understand why GLRQ retains enzymatic activity with a monomeric structure, we carried out molecular dynamics simulations and analysed the structural changes of the catalytic machinery. Four simulation systems were constructed (Table 1): the wild type (WT, PDB ID 1UK2), the inactive monomer R298A (Monomer, PDB ID 2QCY), the GLRQ mutant structure built from the inactive monomer (MutantM), and the GLRQ mutant structure built from the active monomer of the wild-type structure (MutantW). We used several criteria to quantify the catalytic machinery of the enzyme, as in previous studies on 3CLpro^16^,^17^.

#### The catalytic dyad

His41 and Cys145 are two residues of 3CLpro that are directly involved in catalysis^20^. The distance between His41 and Cys145 is critical for the maintenance of their hydrogen bond, which is one stabilizing factor of the catalytic site. We monitored the formation of hydrogen bonds between these two residues in the trajectories (Table 1). The percentages of hydrogen bond formation in the MutantM and MutantW systems were 4.8% and 3.3%, respectively. These percentages were larger than that observed in the Monomer system (1.9%) but smaller than those observed in the WT system (5.2% and 15.6% for the first and second chains, respectively).

#### The Y-X-H motif

The Y-X-H motif contains a hydrogen bond between the OH atom of Tyr161 and the ND1 atom of His163. This motif stabilizes the P1 substrate binding site and is conserved in the main protease of coronaviruses^3^. The percentages of hydrogen bond formation in the MutantM (0.6%) and MutantW (24.8%) systems fall between those observed for the WT (88.3% and 2.0% for the first and second chains, respectively) and the Monomer (0.1%) systems (Table 1).

#### Hydrophobic packing of His163 and Phe140

**Figure 6:**
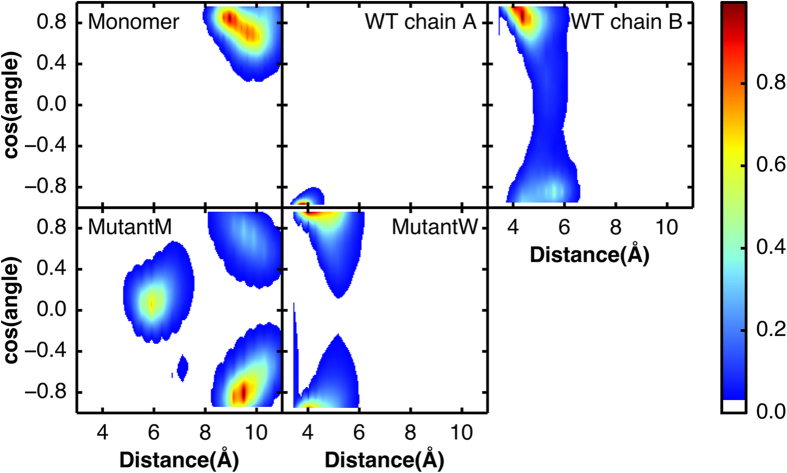
The potential of the mean force of the hydrophobic packing between His163 and Phe140 under different simulation systems. The potential of the mean force (in *k*_B_T) is plotted as a function of the cosine of the dihedral angle between the imidazole ring of His163 and the phenyl ring of Phe140 versus the distance between these two residues (in Å).

The hydrophobic packing of His163 and Phe140 is another stabilizing factor of the substrate P1 binding site of 3CLpro. We analysed the packing between these two residues using two measurements: the distance and the cosine of the dihedral angle between the imidazole ring of His163 and the phenyl ring of Phe140 (Fig. 6). In the Monomer system, the distance between His163 and Phe140 is approximately 9 Å. In the first chain of the WT system (WT Chain A), the side chains of His163 and Phe140 are well packed. The distance is approximately 4 Å and the cosine is close to −1, which means that the rings adopt a face-to-face conformation. These two conformations observed in the Monomer system and WT Chain A represent the inactive and active state of the catalytic site, respectively. In the second chain of the WT system (WT Chain B), the distance is approximately 5 Å and the cosine is shifted to 1. The distribution in MutantW is between the distributions in WT Chains A and B, with two minima at cosine values of −1 and 1, respectively. Although the MutantM system originates from the Monomer system, the MutantM system has a local minimum at a distance of 6 Å and a cosine of 0. This minimum may represent an intermediate state of the inactive-active transition. These results indicate that the conformations of His163 and Phe140 in the mutants fall between the active and inactive state.

#### Secondary structure of Ser139-Phe140-Leu/Thr141

**Figure 7:**
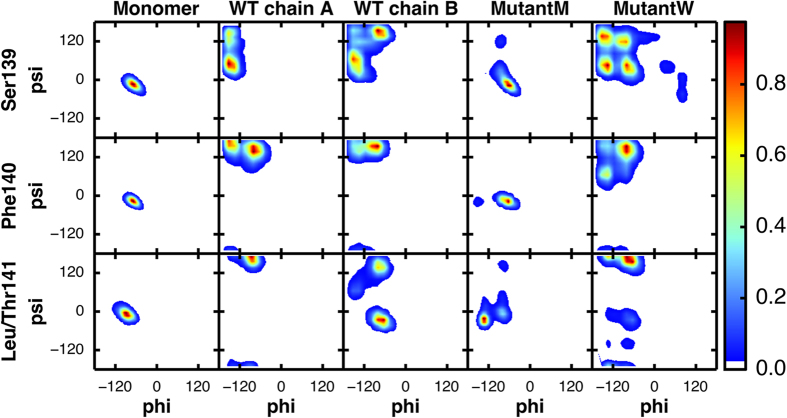
Normalized density plot of the phi/psi dihedrals for residues Ser139-Phe140-Leu/Thr141. Phi vs. psi dihedrals are plotted for each of the residues (Ser139, Phe140, Leu/Thr141) for each of the five systems.

In the inactive structure of the enzyme, the catalytic site collapses due to the formation of a 3_10_-helix at the Ser139-Phe140-Leu141 residues^12^. To determine whether the L141T mutation disrupts this helix, we compared the backbone dihedrals (ϕ, ψ) of these residues (Fig. 7). In the Monomer system, the 3_10_-helix was well preserved. In WT Chain A, however, all residues had non-helical conformations. In WT Chain B, L141 had a stable conformation in the helical region. The conformational distributions of WT Chain A and WT Chain B confirm results from previous studies, which have reported that only one protomer in the dimer is active. In the MutantM system, the helical conformations of S139 and T141 were partially disrupted, showing a small population in the β-region around (ϕ, ψ) = (−90, 120). In the MutantW system, which originated from the non-helical conformation, a sub-stable helical conformation of residue T141 was observed. These results suggest that the L141T mutation partially disrupts the 3_10_-helix.

Taken together, the simulation results show that in GLRQ, the mutation of Leu to Thr partially restores the correct catalytic conformation as designed.

## Discussion

Previous studies using molecular dynamics simulations and hybrid protein experiments have shown that only one protomer of the 3CLpro dimer is active^17^. The reason why nature uses this dimer-monomer combination of activity regulation is unknown but has received much attention. In the present study, we successfully turned SARS-3CLpro into an active monomeric enzyme with four mutations: G11A, L141T, R298A, and Q299A. Three of the mutations, G11A, R298A, and Q299A, were used to maintain a stable monomeric structure. The fourth mutation, L141T, was used to disrupt the 3_10_-helix structure that is formed by the Ser139-Leu141 tri-peptide in the inactive monomer and restore the active conformation.

The active monomer of the GLRQ mutant maintains a stable monomer structure as shown by analytical gel filtration and analytical ultracentrifugation analysis. GLRQ maintains its monomer structure in the presence of a substrate analogue. In contrast to the wild-type enzyme, *k*_cat_/*K*_*m*_ of GLRQ did not change with changes in enzyme concentration. These experimental results verify that GLRQ is an active monomer. These results also confirm that the flexibility of the Ser139-Leu141 loop is essential for 3CLpro enzyme activity.

The short 3_10_-helix formed by Ser139-Leu141, which twisted the oxyanion hole in the monomer structure, has also been found in the inactive 3C protease (3Cpro) mutant of the Hepatitis A virus (HAV), which belongs to the picornavirus family. The wild-type HAV-3Cpro is active as a monomer^24^. Residues 169–172 of HAV-3Cpro, which normally form an oxyanion pocket, adopt a 3_10_-helical conformation in the crystal structure of the inactive C172A mutant. Results from the mutant C172A of HAV-3Cpro demonstrate that the enzyme is active when the loop that forms the oxyanion hole is in the correct conformation, regardless of whether the enzyme molecules form a stable dimer or remain monomeric.

Our molecular dynamics simulations showed that Ser139-Leu141 maintains a stable 3_10_-helix conformation in the inactive monomer structure and a well-defined loop conformation in the active protomer of the dimer structure. For the mutant GLRQ, the loop conformation and active site structure transitioned between inactive and active structures regardless of whether the simulation started from an inactive conformation or an active conformation. Because the three mutations, G11A, R298A, and Q299A, stabilize the monomer structure, the only mutation in the Ser139-Leu141 loop, L141T, was deduced to be the major driving force that destabilizes the 3_10_-helix towards the active loop conformation. This single mutation may not be sufficient to achieve a highly active enzyme, however. More mutations may be necessary to increase the activity of GLRQ. The successful design of an active monomer of SARS-3CLpro nevertheless demonstrates the importance of the Ser139-Leu141 loop conformation, as the loop 3_10_-helix transition serves as a switch for enzyme activity.

Although SARS-3CLpro uses the dimer structure to maintain its enzyme activity, our study shows that the monomer can also be evolved into an active enzyme via mutations. Why does nature select the dimer structure as the active form for SARS-3CLpro when multiple strategies for enzyme activity regulation are available? Our current study provides more evidence that the virus needs a main proteinase to process its viral polyprotein at the appropriate time. A previous study has shown that the substrate can enhance SARS-3CLpro dimerization^21^. During the viral replication process, the enzyme modulates its activity to control the digestion process as the amount of polyproteins change via dimer association and dissociation^22^. In addition to maintaining enzyme activity, SARS-3CLpro also needs to tune its activity according to the substrate concentration. The SARS virus may use this strategy to simultaneously prepare proteins that are necessary for assembly.

In conclusion, by tuning the conformation flexibility of an active site loop, we have successfully designed an active monomer mutant of SARS-3CLpro. The present work not only serves as a model system to study the regulatory mechanism of enzyme activity, but also facilitates the discovery of anti-viral treatments and the response to emerging coronaviruses such as MERS-CoV^25^.

## Methods

### Site-directed mutagenesis, expression, purification, and activity detection

The mutants G11A/L141T/R298A (GRQ) and G11A/L141T/R298A/Q299A (GLRQ) of SARS-3CLpro were generated with the QuikChange site-directed mutagenesis kit (Stratagene, La Jolla, CA, USA) using pET 3CLP-21h^20^ or its derivatives as templates. The primers for all mutations are listed in Supplemental Table S3. The mutation was verified by DNA sequencing (Invitrogen, Beijing, China). All resulting plasmids were transformed into *E. coli* BL21(DE3) cells for expression. The recombinant SARS-3CLpro and its mutants were prepared using ammonium sulfate fractional precipitation and chromatography, as previously described^20^.

Enzyme activity was determined using a colorimetric substrate, Thr-Ser-Ala-Val-Leu-Gln-pNA, as previously reported^20^. Substrate (200 μM) was mixed with 18 μM GRQ, 1.8 μM GLRQ, or 1.8 μM 3CLpro at 37 °C to compare their reaction rates. *K*_*m*_ and *k*_cat_ of GLRQ were studied by adding the preheated substrate to a reaction mixture to obtain a final concentration of 40.6 μM GLRQ and 100–400 μM substrate. Values of *K*_*m*_ and *k*_cat_ were calculated by the Lineweaver-Burk plot. Enzyme activity dependence of GLRQ at different concentrations was measured by fixing peptide-pNA substrate at 200 μM and varying the enzyme concentration from 1.85 to 28.7 μM.

### Inhibition assay

The isatin derivative, 1-(2-naphthlmethyl) isatin-5-carboxamide (**5f**)^26^, is known to inhibit SARS-3CLpro. **5f** was dissolved in dimethyl sulfoxide (DMSO) to test inhibition efficiency towards GLRQ. GLRQ (18.5 μM) and **5f** (160 μM) or control 2% DMSO were pre-incubated in 40 mM of phosphate buffer (pH 7.3) at 37 °C for 5 min, and then 200 μM of the substrate (Thr-Ser-Ala-Val-Leu-Gln-pNA) was added to the mixture and allowed to react for 750 s.

### Analytic ultracentrifugation analysis

Sedimentation velocity experiments were conducted with a Beckman Optima XLA analytical ultracentrifuge using a previously reported procedure^22^. One mg/ml 3CLpro, GRQ, or GLRQ was loaded into the double-sector centrepieces. Absorbance scans were collected at 286 nm with a speed of 56,000 rpm. In addition, 5 μM GLRQ in 40 mM of phosphate buffer (pH 7.3) with or without 10 μM of the substrate-like inhibitor **5f**^26^ were also loaded and analysed at 240 nm with the same speed. Three μM 3CLpro with 0 or 1 μM **5f** was also scanned at 230 nm with the same speed and temperature as the controls^21^.

### Analytic gel filtration analysis

The oligomeric states of GRQ and GLRQ were tested on the Superdex 75 HR 10/300 GL column using previously reported procedures^22^. The purified protein was loaded onto the column at two different concentrations: 4 mg/ml and 0.52 mg/ml for GRQ and 4 mg/ml and 0.58 mg/ml for GLRQ. Four gel filtration molecular weight markers, albumin from bovine serum (5 mg/ml), ovalbumin (2.5 mg/ml), carbonic anhydrase (1.5 mg/ml), and cytochrome C (1 mg/ml), were also loaded onto the column and calibrated to determine a standard calibration curve based on the retention volumes (Supplemental Fig. S2 and Table S1). Two concentrations of 3CLpro (4 mg/ml and 0.52 mg/ml) were also analysed on the Superdex75 HR as controls.

### Molecular dynamics simulations

Simulations were conducted with the Gromacs package^27^ using the OPLS-AA force field^28^. The simple point charge (SPC) model of water was used to solvate the protein in a periodic dodecahedron box extending 10 Å from the nearest protein atom. The solvated system then was neutralized with Na^+^ and Cl^−^ ions, minimized by the steepest descent method (5000 steps), and equilibrated with a 100-ps constant volume (NVT) simulation. The production runs were conducted in the constant pressure ensemble (NPT). The temperature was set to 300 K and controlled with a modified Berendsen thermostat^29^. Long-range electrostatic interactions were treated with the particle-mesh Ewald method^30^. The pressure was coupled to 1 bar with the Parrinello-Rahman method^31^. All bond lengths were constrained with the LINear Constraint Solver (LINCS) algorithm^27^. A cut-off of 10 Å was used to calculate short-range van der Waals and electrostatic interactions. The time step was 2 fs and the trajectories were saved every 20 ps.

## Additional Information

**How to cite this article**: Li, C. *et al.* Conformational Flexibility of a Short Loop near the Active Site of the SARS-3CLpro is Essential to Maintain Catalytic Activity. *Sci. Rep.* **6**, 20918; doi: 10.1038/srep20918 (2016).

## Supplementary Material

Supplementary Information

## Figures and Tables

**Table 1:**
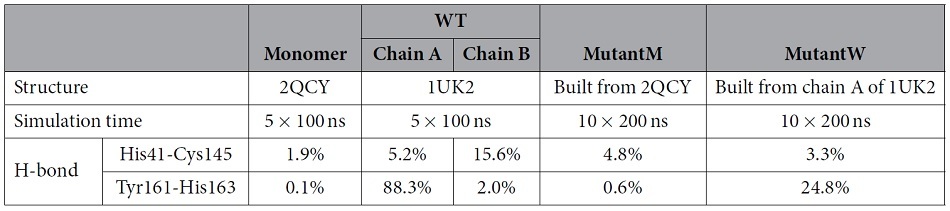
Used systems and percentage of hydrogen bonds formed in the molecular dynamics simulations.
