# Supplementary material for: Conformational Flexibility of a Short Loop near the Active Site of the SARS-3CLpro is Essential to Maintain Catalytic Activity
Source: Sci Rep. 2016 Feb 16;6:20918. doi: 10.1038/srep20918 (PMC4754693; doi:10.1038/srep20918)
Supplement: Supplementary Information [file srep20918-s1.doc]

**Supplementary information for**

**Conformational Flexibility of a Short Loop near the Active Site of the SARS-3CLpro** **is Essential to Maintain Catalytic Activity**

Chunmei Li^1,2,+^, Xin Teng^1,+^, Yifei Qi^2,+^, Bo Tang^1^, Hailing Shi^1^, Xiaomin Ma^2^, Luhua Lai^1,2^,*

^1^ BNLMS, State Key Laboratory for Structural Chemistry of Unstable and Stable Species, College of Chemistry and Molecular Engineering, Peking University, Beijing, 100871, China

^2^ Center for Quantitative Biology, Peking University, Beijing, 100871, China

* Corresponding author: [lhlai@pku.edu.cn](mailto:lhlai@pku.edu.cn)

^+^ These authors contributed equally to this work.


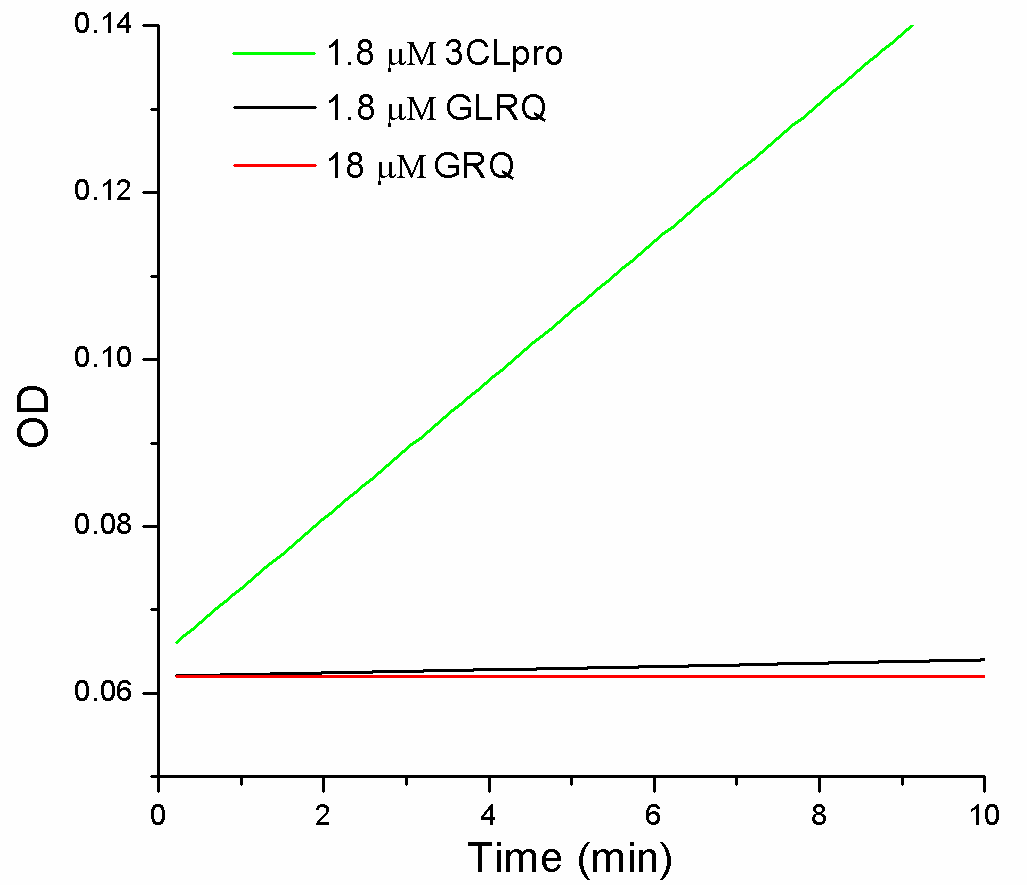


**Figure S1.** Enzyme activity comparison of GRQ, GLRQ and 3CLpro. 18 µM GRQ, 1.8 µM GLRQ and 1.8 µM 3CLpro have reacted with 200 µM colorimetric substrate, Thr-Ser-Ala-Val-Leu-Gln-pNA, at 37 ^○^C, respectively. The velocity of GRQ, GLRQ amd 3CLpro have been shown in red line, black line and green line, respectively.


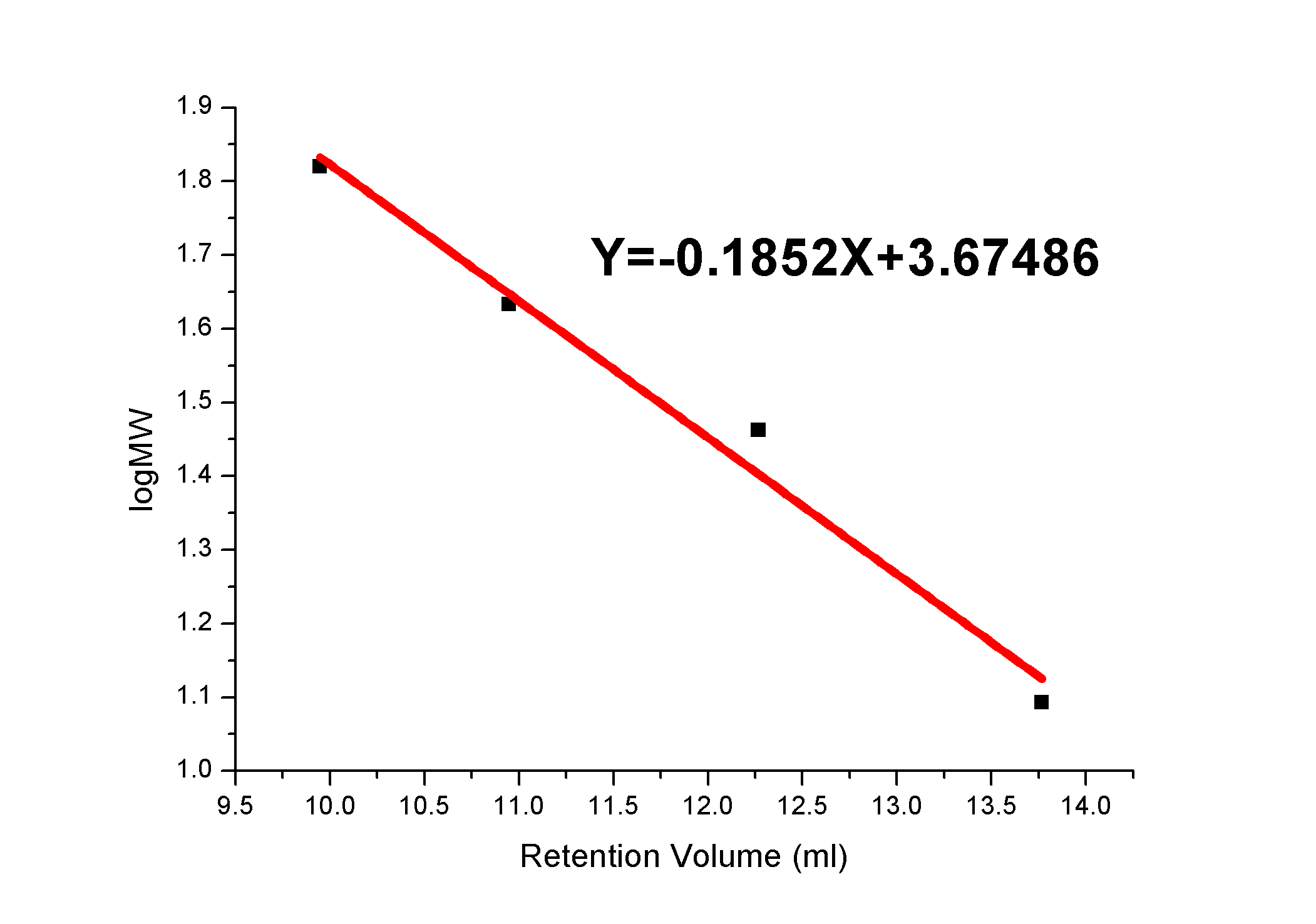


**Figure S2.** Standard curve. The standard curve was calculated by plotting the logarithm of the standard protein molecular weight markers versus the retention volume (Table S1).


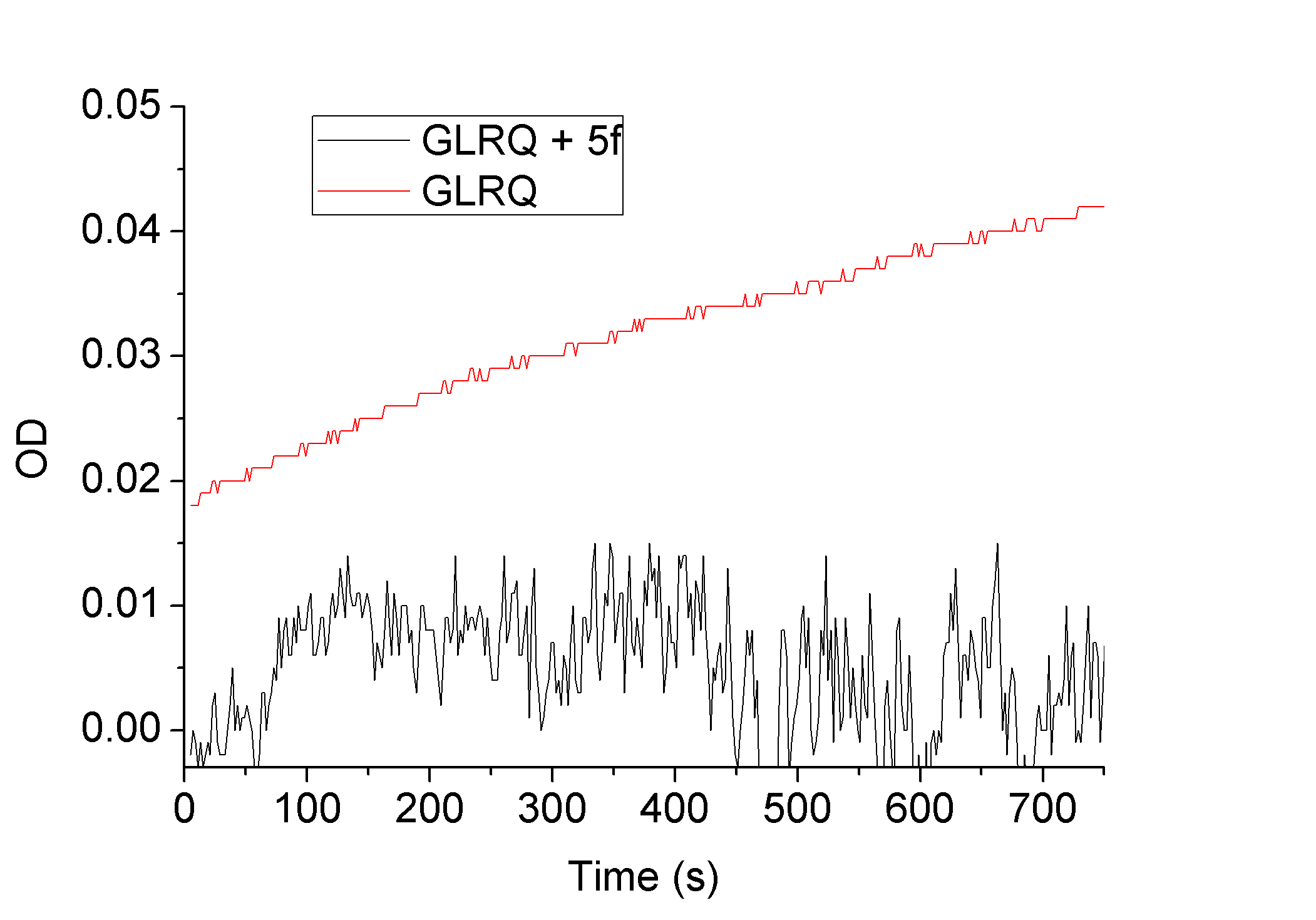


**Figure S3.** The inhibition assay of **5f** on GLRQ. The compound concentration was 160 µM, and the enzyme (GLRQ) concentration was 18.5 µM. The red line represents the reaction profile of 18.5 µM GLRQ with 200 pNA substrate in 40 mM PBS. The black line represents the reaction profile of 18.5 µM GLRQ with 200 µM pNA substrate and 160 µM **5f** in 40 mM PBS.


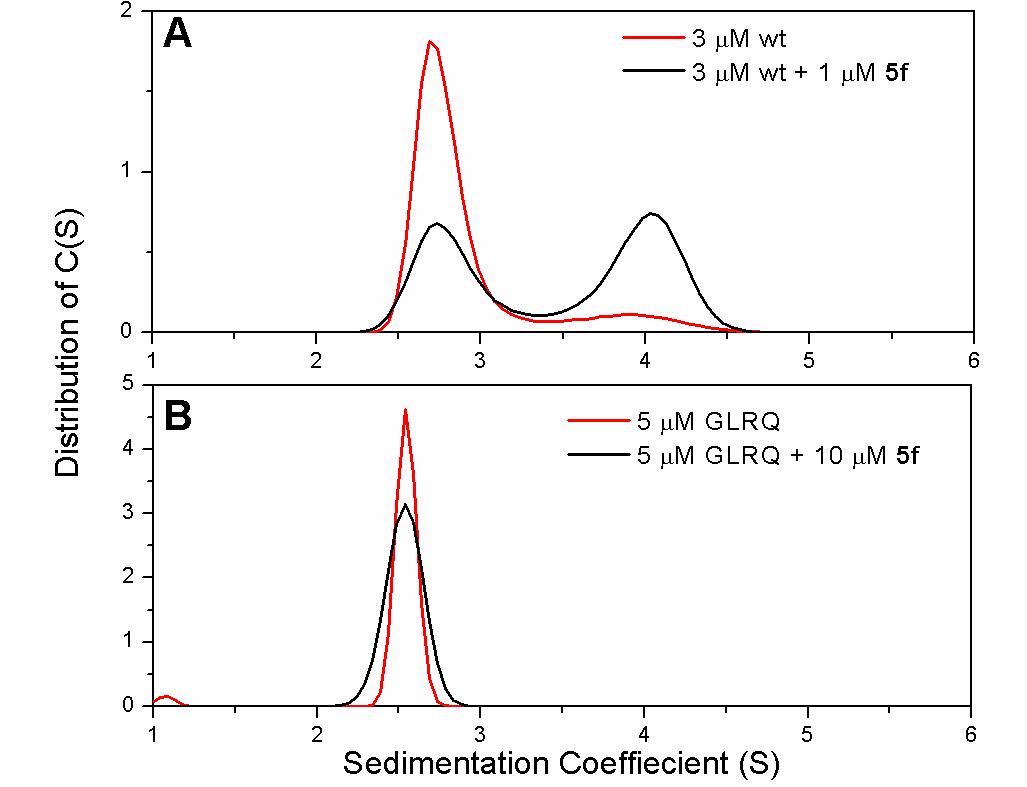


**Figure S4**. The sedimentation coefficient distribution of 3CLpro (A) and GLRQ (B) with isatin derivative **5f**. An apparent dimer peak at 4.0 s was induced by 1 µM **5f** for 3 µM 3CLpro but not for GLRQ.

| Name of marker | MW (kDa) | Log MW | Retention Volume (ml) |
| --- | --- | --- | --- |
| albumin from bovine serum | 66 | 1.819 | 9.947 |
| ovalbumin | 43 | 1.633 | 10.948 |
| carbonic anhydrase | 29 | 1.462 | 12.268 |
| cytochrome c | 12.4 | 1.093 | 13.767 |

**Table S1.** The retention volume of all the gel filtration molecular weight markers.

| Proteins | Concentration (mg/ml) | Retention volume (ml) | Putative MW (kDa) | Theoretical MW (kDa) |
| --- | --- | --- | --- | --- |
| GRQ | 0.52 | 12.021 | 28.1 | 33.7 |
|  | 4.00 | 12.021 | 28.1 |  |
| GLRQ | 0.58 | 12.039 | 27.9 |  |
|  | 4.00 | 12.029 | 28.0 |  |
| 3CLpro | 0.52 | 11.884 | 29.8 |  |
|  | 4.00 | 11.490 | 35.2 |  |

**Table S2.** Retention volumes of GRQ, GLRQ, and 3CLpro at different concentrations and their putative molecular weights (MW) as determined from the calculated standard curve.

| Primer | Oligonucleotide sequence (5’-3’) |
| --- | --- |
| G11A-F | GGCATTCCCGTCAGCTAAAGTTGAAGGGTGC |
| G11A-R | GCACCCTTCAACTTTAGCTGACGGGAATGCC |
| L141T-F | CCATTAAAGGTTCTTTCACTAATGGATCATGTG |
| L141T-R | CACATGATCCATTAGTGAAAGAACCTTTAATGG |
| R298A/Q299A-F | ACACCATTTGATGTTGTTGCAGCATGCTCTGGTGTTACCTTC |
| R298A/Q299A-R | GAAGGTAACACCAGAGCATGCTGCAACAACATCAAATGGTGT |

**Table S3.** The primers used to generate the SARS-3CLpro mutants.
